# Commentary: Heart–brain interaction in cardiogenic dementia: pathophysiology and therapeutic potential

**DOI:** 10.3389/fcvm.2024.1479675

**Published:** 2025-01-10

**Authors:** Bupe Amon Kyelu, Phillip Taderera Bwititi, Kate Kauter, Ezekiel Uba Nwose

**Affiliations:** ^1^ School of Health and Medical Science, University of Southern Queensland, Toowoomba, QLD, Australia; ^2^ School of Dentistry and Biomedical Sciences, Charles Sturt University, Wagga Wagga, NSW, Australia

**Keywords:** mental health, cardiovascular disease, pathology evaluation, routine laboratory indicators, evidence-base medicine, blood viscosity

A commentary on Commentary: Heart-brain interaction in cardiogenic dementia: pathophysiology and therapeutic potential By Liu J, Xiao G, Liang Y, He S, Lyu M and Zhu Y (2024). Front. Cardiovasc. Med. 11:1304864. doi: 10.3389/fcvm.2024.1304864

## Introduction

In the paper, “Heart-brain interaction in cardiogenic dementia: pathophysiology and therapeutic potential” (1), it is acknowledged that heart failure (HF) may constitute the greatest burden of cardiovascular disease (CVD), which is a major public health issue globally. The authors draw attention to CVD physiology as well as the implications for cardiovascular medicine and also articulate and update three themes – risk factors, effects on cerebral blood flow (CBF) and preventive management – of cognitive impairment theory and mental health areas with a focus on dementia.

From a laboratory medicine perspective, the term “blood flow” is mentioned 5 times, and “platelet” 3 times. However, neither “blood test” for blood flow nor “viscosity” were indicated. Hence, from laboratory or evidence-base medicine perspective, there is opportunity to advance blood viscosity concept in dementia management. In this paper, the risk factors (atrial fibrillation, coronary artery disease, heart failure, myocardial infarction, and valvular heart disease) of cardiogenic dementia that can be associated with increased blood viscosity, are discussed. Further, the authors have elucidated on the mechanism to involve hypoperfusion and oxidative stress both leading to hyperviscosity, as well as prevention therapies to reduce blood viscosity (Table 1).

**Table 1:** Rearticulation of blood viscosity in cardiogenic dementia pathophysiology.

Theme	Factors	Effect on blood flow
Mechanism	Hypoperfusion	Increased oxidative stress leads to hyperviscosity, which complicates CBF
	Neurohormonal rennin-angiotensin system	Reduced CBF and exacerbated oxidative stress which would be indicated by hyperviscosity
Prevention	Statins	Reduced lipidaemia leads to low blood viscosity
	Anticoagulant	Apparently improves blood flow

## Antiplatelet management of hyperviscosity and implication for laboratory testing

Hyperviscosity constitutes a cardiovascular phenomenon, which connotes stasis (2), and the management of stasis in clinical practice involves antiplatelet drugs such as aspirin (3, 4). What is probably arguable is the practice of evidence-base monitoring of the therapeutic effectiveness. That is, the extent that the antiplatelet drugs such as aspirin and clopidogrel reduce the hyperviscosity being managed by laboratory monitoring methods. This therefore brings to the fore the concept of measuring blood viscosity in clinical practice. Perhaps, a second question is awareness of availability and utility of blood viscosity measurement.

There have been alternative tests for plasma viscosity and whole blood viscosity (5–8), which were limited to reference laboratory, but are no longer available. There is also the option of estimated or extrapolated whole blood viscosity (eWBV) (8), which is applicable in cardiovascular medicine (9, 10), including metabolic syndrome and mental health services but not yet adopted in clinical practice presumably due to lack of awareness.

## Discussion: antiplatelet drug monitoring in cardiovascular medicine

This commentary advances the discourse of cardiogenic dementia to highlight a gap in knowledge and practice of mental health service. The implication of hyperviscosity in the pathophysiology and therapeutic potentials (1), means that laboratory evidence-base determination would need to studied to understand the course of hyperviscosity in pathophysiology and its response to treatment of various disorders.

It is pertinent to emphasize that the option of eWBV has been known for a long time and applicable in mental health management (8), and investigation of possible cardiovascular outcomes (5, 10), as well as antiplatelet therapy monitoring in diabetes (11). That is, blood viscosity evaluation including the eWBV option, is well established in clinical research, but there has been retrogress in clinical practice (9). Cardiogenic dementia has been recognised for over three decades (12), but there has been a lack of movement/research, which is evidenced by dearth or clinical trial or meta-analysis on the subject.

To expatiate a bit more on antiplatelet drug monitoring: it is noteworthy that patients deemed for antiplatelet therapy are supposed to be assessed against risk of bleeding prior to treatment (13). However, this is rarely done, most probably because the pathology is deemed unavailable, whereas eWBV is a useful tool (11). The methodology of eWBV is simple algorithm using haematocrit (packed cell volume) and serum protein from routine haematology and liver function tests, respectively (10, 11). Clinical guidelines for practical implementation are essentially the two opposite sides of haemostasis imbalance which often requires monitoring (3). That is, where bleeding risk or thrombosis would be identified with hypo-viscosity or hyper-viscosity, respectively.

Therefore, it is important to note that Liu and colleagues advanced the discourse on cardiogenic pathophysiology, and research can now review the aspect of laboratory evidence-base medicine. Further, the idea of monitoring rheological variables with laboratory tests is known but often limited to routine haematology including blood cell counts (14). This commentary advances the concept eWBV, which is at no additional “cost-of-production” to the health service providers.

It is also pertinent to emphasize that blood viscosity test in clinical practice used to be performed albeit in reference laboratories thus, limited access, by most clinicians, especially the remote and rural healthcare providers. This has resulted in under-utilisation of this important diagnostic test that is affordable since it can be estimated from routine lab results (9). Current testing provides a valid result only if immediate access to pathology services is available since transporting the sample for long distance can result in the deterioration. The newly-developed algorithm for estimating WBV from haematocrit and total serum protein levels, which are routine blood tests, offers ready access to this important measure of WBV.

## Implications for health services research

This new process for assessing WBV using results from routine blood tests such as haematocrit and total serum protein can be accessed without using advanced testing. This means it can be used for various common diseases that are associated with changes in WBV, such as stress, with outpatient mental health and alcohol & other drugs services, diabetes mellitus, cardiovascular and kidney diseases (15). Further, this medical assessment does not require clients to be hospitalized or to travel to reference facility. Requests for WBV testing e.g., for a mental health client's evaluation of stress levels or for an individual living with cardiovascular disease can be done by a GP or primary healthcare provider (16–19), without the client needing to travel.

There has been the question whether blood flow changes with mood (20) and studies have reported that this can be the case. For instance, it is established that physical restraint of client can cause blood flow stasis leading to development of thrombosis complications (21). Therefore, suffice to conclude that there is reasonable justification to advance the laboratory evidence-base of cardiogenic mental health.
